# Trends in Prehospital Visits as a Cause of Delayed Admission in Korean Stroke Patients over a 10-Year Period: A National Health Insurance Claims Data Study

**DOI:** 10.31083/j.rcm2403083

**Published:** 2023-03-06

**Authors:** Jinyoung Shin, Hyeongsu Kim, Ho Jin Jeong, Jeehye Lee, Jusun Moon, Kwang-Pil Ko, Youngtaek Kim

**Affiliations:** ^1^Department of Family Medicine, Konkuk University School of Medicine, 05030 Seoul, Republic of Korea; ^2^Department of Preventive Medicine, Konkuk University School of Medicine, 05030 Seoul, Republic of Korea; ^3^National Emergency Medical Center, National Medical Center, 04564 Seoul, Republic of Korea; ^4^Department of Neurology, National Medical Center, 04564 Seoul, Republic of Korea; ^5^Clinical Preventive Medicine Center, Seoul National University Bundang Hospital, 13620 Gyeonggi-do, Republic of Korea; ^6^Department of Preventive Medicine, Chungnam National University Hospital, 35015 Daejeon, Republic of Korea

**Keywords:** stroke, trends, delayed diagnosis, administrative claims, healthcare

## Abstract

**Background:**

A prehospital delay from symptom onset to hospital arrival resulted in stroke-related complications or in-hospital deaths in acute stroke patients. We aimed to investigate trends in prehospital visits as a cause of prehospital delay using data from the Korean Health Insurance Service.

**Methods:**

This nationwide, population-based, retrospective cohort study included 524,524 newly-diagnosed stroke patients admitted via the emergency departments of secondary and tertiary hospitals. We obtained the prehospital visits rate from 2010 to 2019 and identified the related characteristics.

**Results:**

Prehospital visits were observed in 111,465 patients (21.3%). The prehospital visits rate decreased from 25.1% in 2010 to 17.8% in 2019, but the number of patients increased from 11,255 cases in 2010 to 11,747 cases in 2019. Fortunately, the rate of delayed admission for more than one day decreased from 26.7% to 21.3%. However, 10.4% of patients were diagnosed more than two days later. Young, females, or patients with higher income status and living in low urbanization areas exhibited a higher rate of prehospital visits.

**Conclusions:**

Prehospital visits in Korean stroke patients decreased from 25.1% in 2010 to 17.8% in 2019. However, more than 10,000 patients still visited other medical institutions before admission to treatment.

## 1. Introduction 

A prolonged delay from the stroke onset to hospital arrival decreases the efficacy of time-dependent treatments and results in a greater frequency of stroke-related complications or in-hospital deaths [1, 2, 3]. The prolonged delay is classified into a prehospital delay from symptom onset to arrival at the emergency department (ED) and an in-hospital delay between the patient’s arrival at the hospital and treatment initiation [1, 4]. A prehospital delay (89.2%) constitutes the most significant proportion of total delay time compared to the in-hospital delay [5], despite a 6.0% annual decline of prehospital delay in hours per year among the 123 studies between 1981 and 2007 [2]. Awareness among the patient or bystander that the initial symptom was stroke-related and transportation using an emergency medical service (EMS) were associated with an early arrival to the hospital [6, 7, 8], while living alone or being alone at the onset of symptoms and referral from other hospitals are commonly linked to delayed arrival [7, 8, 9, 10].

After launching the acute stroke quality assessment program as an external audit by the Health Insurance Review and Assessment Service beginning in 2007, defect-free stroke care quality has improved in Korea [11]. Korean patients can choose their health care providers and institutions because of the low barrier to medical service use. Among acute stroke patients, about half of them are directed to primary care clinics or other healthcare professionals without using EMS [12]. Knowing the trends in prehospital visits using a representative and extensive database is necessary to aid in policy-making to reduce prehospital delays. Several studies have been conducted via a patient survey or medical records review [13, 14, 15], but did not directly examine annual trends.

Using a national database, this study aimed to evaluate trends and characteristics in the prehospital visit of newly-diagnosed stroke patients using a national database.

## 2. Materials and Methods

### 2.1 Study Design

This study sought to examine the 10-year trend in prehospital visits using the National Health Information Database (NHID) including health care utilization, health screening, socio-demographic variables, and mortality from the National Health Insurance Service (NHIS) from 2010 to 2019. The NHID covers the entire Korean population (97.2% of NHIS and 2.8% of medical aid). The eligibility database includes information about income-based insurance contributions, demographic variables, and date of death. The national health screening database provides information on health behaviors and bio-clinical variables. The NHID includes information on records of inpatient and outpatient usage and prescription. The long-term care insurance database provides information about activities of daily living and service grades. The health care provider database includes data about the types of institutions, human resources, and equipment. In the NHID, de-identified join keys are used to interlink these databases. The NHIS provides the platform for customized retrospective cohort data [16].

New onset of stroke diagnosis was identified by the inclusion of the following International Classification of Diseases, 10th revision (ICD-10) codes in NHIS data: I60 (subarachnoid hemorrhage), I61 (intracerebral hemorrhage), I62 (other non-traumatic intracranial hemorrhage), I63 (cerebral infarction), or I64 (stroke not specified as hemorrhage or infarction) without a previous diagnosis in the three years before.

### 2.2 Study Participants

**Fig. 1.**
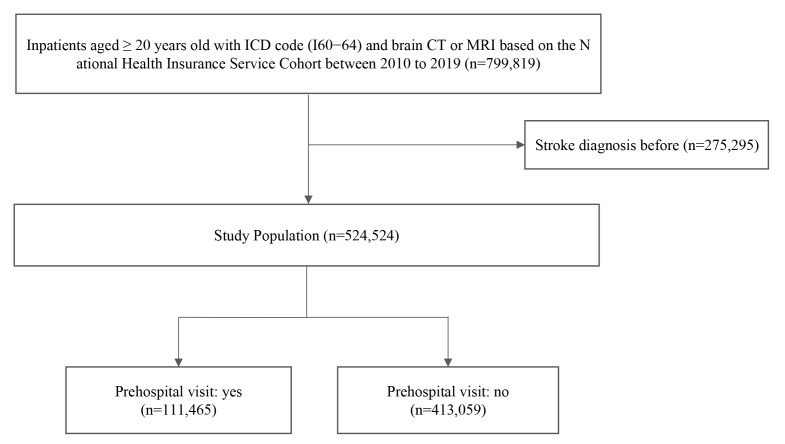
**Flow chart of study population selection**.

Prehospital visits of acute stroke patients aged at least 20 years old and admitted via the EDs of secondary and tertiary hospitals with brain computer tomography (CT) or magnetic resonance image (MRI) were assessed from January 1, 2010, to December 31, 2019 (n = 799,819). After excluding 275,295 subjects with a previous stroke diagnosis, we finally analyzed the data of 524,524 participants (Fig. 1).

A prehospital visit was defined as visiting a medical institution to treat conditions of I60 to I64, I65 (occlusion and stenosis of vertebral artery), or I66 (occlusion and stenosis of middle cerebral artery), I67 (other cerebrovascular diseases), or I68 (cerebrovascular disorder in diseases classified elsewhere), regardless of whether the patient had visited a primary, secondary, or tertiary hospital. According to the occurrence of a prehospital visit, we divided patients into two groups: a prehospital visit group (n = 111,465) and a no-hospital-visit group (n = 413,059).

### 2.3 Study Variables

Age was calculated from the participant’s birth year from the index data as the date of the first diagnosis of stroke. Income status was obtained based on insurance status and was divided into six groups (medical aid and lowest quintile to highest quintile). Residential area was classified into two groups; the “high urbanization” area was defined as including administrative districts with a tertiary hospital or a stroke care unit, while areas other than these were classified as “low urbanization”.

### 2.4 Statistical Analyses

Age is presented as mean with standard deviation values and categorical variables for the subgroup analysis, such as 20 to 44 years old, 45 to 64 years old, and at least 65 years old. We compared the characteristics of study subjects between the prehospital and no-hospital-visit groups using the t-test and chi-squared test. We calculated the annual rate of prehospital visits from 2010 to 2019 according to three groups, i.e., one, two, and at least three prehospital visits, respectively. We presented the 10-year trends in prehospital visits according to age group, sex, insurance unit, and residence. The time intervals between the prehospital visit and the day of final admission for treatment were obtained by date because of claims data. We presented the first visit time among the patients with more than two visits. All analyses were performed using SAS version 9.4 (SAS Institute Inc., Cary, NC, USA), and *p *< 0.05 was considered to indicate statistical significance.

### 2.5 Ethics Statement 

The present study was conducted following the principles of the Declaration of Helsinki. This study was approved by the institutional review board of the Clinical Research Ethics Committee of Konkuk University, Seoul, Korea (approval no. 7001355-202105-E-140), which also waived the requirement for informed consent because this study did not include identifying individuals and employed previously collected data gathered from the general public. It was also approved in a review by the internal committee of NHIS (NHIS study no. NHIS-2021-1-479).

## 3. Results

### 3.1 Demographic Characteristics

The characteristics of the 524,524 stroke patients enrolled in this study are shown in Table 1. A total of 111,465 patients (21.3%) were found to have visited any hospital with stroke-related ICD codes before admission through ED. The mean age of the study participants was 64.7 ± 14.5 years old. Younger and female patients were more likely to be classified into the prehospital visit group (*p *< 0.001 and *p *= 0.012, respectively). Patients who resided in high urban areas were more likely to be in the no-hospital-visit group (*p *< 0.001). Considering income status, the prehospital and no-hospital-visit group rates differed (*p *< 0.001).

**Table 1.** **Demographic and clinical characteristics of study subjects according to prehospital visits**.

		Total	Prehospital visit		*p*-value
			Yes	No	
N (%)		524,524	111,465 (21.3)	413,059 (78.7)	
Age, mean ± SD		64.7 ± 14.5	63.7 ± 14.3	64.9 ± 14.5	<0.001
	20–44	46,790 (8.9)	10,945 (23.4)	35,845 (76.6)	<0.001
	45–64	201,457 (38.4)	44,632 (22.2)	156,825 (77.8)	
	≥65	276,277 (52.7)	55,888 (20.2)	220,389 (79.8)	
Sex					0.012
	Male	290,098 (55.3)	61,279 (21.1)	228,819 (78.9)	
	Female	234,426 (44.7)	50,186 (21.4)	184,240 (78.6)	
Income units					<0.001
	Q1	35,553 (6.8)	6697 (18.8)	28,856 (81.2)	
	Q2	84,182 (16.0)	18,044 (21.4)	66,138 (78.6)	
	Q3	71,691 (13.7)	15,695 (21.9)	55,996 (78.1)	
	Q4	83,645 (15.9)	18,767 (22.4)	64,878 (77.5)	
	Q5	102,083 (19.5)	22,288 (21.8)	79,795 (78.2)	
	Q6	147,370 (28.1)	29,974 (20.3)	117,396 (79.7)	
Urbanization					<0.001
	High	330,221 (63.0)	54,920 (16.6)	275,301 (83.4)	
	Low	194,303 (37.0)	56,545 (29.1)	137,758 (70.9)	

Data were shown as n (%) or mean value ± standard deviation (SD). Q1, lowest; Q6, highest. High urbanization area, administrative districts with a tertiary hospital or a stroke care unit; low urbanization area, the other area.

### 3.2 The 10-Year Trends in Prehospital Visits among Korean Stroke Patients

**Fig. 2.**
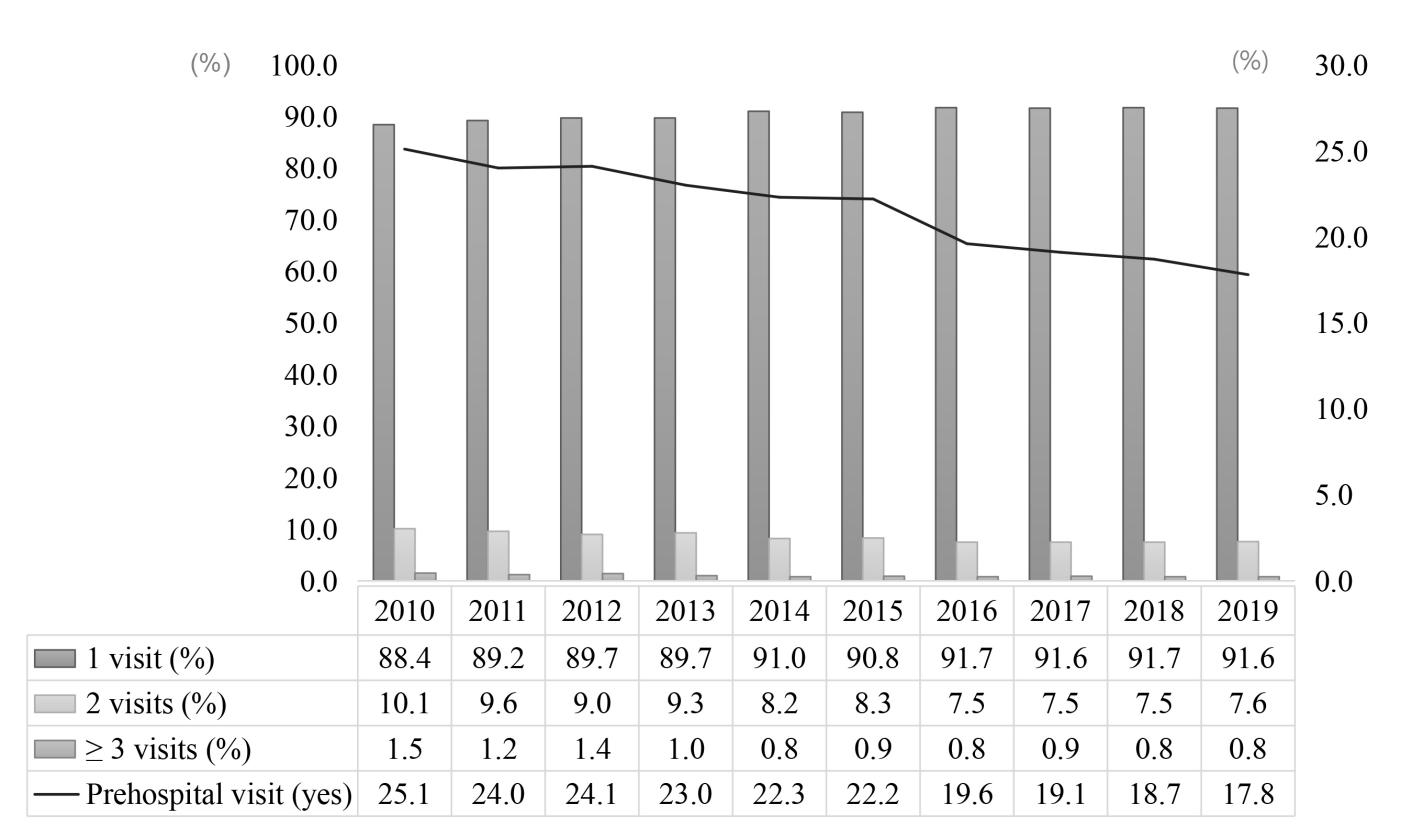
**10-year trends in prehospital visits in newly-diagnosed stroke patients**.

The annual prehospital visits rates continuously decreased over time from 2010 to 2019 (Fig. 2). In 2010, 25.1% of patients (11,255 cases) had visited one or more hospitals before diagnosis. Although the number of patients with prehospital visits slightly increased (11,747 cases), the total rate of prehospital visits decreased to 17.8% in 2019, reflecting an increase in stroke incidence. The rates of two prehospital visits, or at least three, decreased from 2010 to 2019.

Even though the case number of strokes increased rapidly during the observation period, the annual mean rate of prehospital visits continuously decreased, or the number of them did not increase significantly (Table 2). Prehospital visits rate also reduced according to age, sex, income status, and residential area subgroups. During the ten years of this study, younger patients or living in low urban areas showed a higher rate of prehospital visits. However, the higher rate of prehospital visits among female patients observed beginning in 2010 was reversed from 2016 to 2019. In 2010, subjects with higher income status exhibited a higher rate of prehospital visits than those with lower income status; however, the pattern disappeared in recent years.

**Table 2.** **Trends of prehospital visits in stroke patients according to participants characteristics**.

		2010		2011		2012		2013		2014		2015		2016		2017		2018		2019	
		n	%	n	%	n	%	n	%	n	%	n	%	n	%	n	%	n	%	n	%
Total stroke		44,766		45,555		45,253		46,345		47,419		48,595		58,204		60,608		61,842		65,937	
Prehospital visits		11,255	25.1	10,944	24.0	10,915	24.1	10,677	23.0	10,564	22.3	10,795	22.2	11,392	19.6	11,601	19.1	11,575	18.7	11,747	17.8
Age																					
	20–44	1280	29.5	1160	26.4	1137	26.9	1104	25.7	1109	26.0	1043	25.3	1020	19.0	1074	20.1	1029	20.1	989	18.6
	45–64	4505	26.5	4402	25.1	4365	25.2	4289	24.2	4181	23.1	4440	23.7	4533	19.9	4635	19.9	4590	19.4	4692	18.5
	≥65	5470	23.4	5382	22.8	5413	22.9	5284	21.7	5274	21.0	5312	20.6	5839	19.4	5892	18.5	5956	18.0	6066	17.2
Sex																					
	Male	6063	24.6	5907	23.6	5929	23.7	5855	22.8	5747	21.9	5861	21.8	6313	19.7	6415	19.2	6482	18.9	6707	18.3
	Female	5192	25.9	5037	24.6	4986	24.6	4822	23.4	4817	22.7	4934	22.8	5079	19.4	5186	19.1	5093	18.5	5040	17.2
Income status																					
	Q1	1118	23.5	1073	22.8	986	22.0	953	22.0	733	19.6	728	19.0	844	18.2	821	17.3	835	16.5	851	16.0
	Q2	1586	25.0	1648	24.3	1616	24.0	1634	22.5	1661	22.6	1761	22.7	1776	20.0	1796	19.3	1904	19.1	2213	18.7
	Q3	1534	25.8	1501	24.5	1608	25.5	1387	23.2	1510	23.2	1416	22.5	1630	19.8	1675	20.1	1620	19.5	1365	17.6
	Q4	1843	26.4	1797	25.3	1749	24.9	1736	23.8	1758	23.7	1803	23.6	1892	20.7	1981	20.9	1852	19.3	1907	19.0
	Q5	2287	26.7	2106	24.6	2126	24.9	2164	24.3	2030	22.2	2170	23.1	2210	19.4	2203	19.2	2251	19.2	2292	17.9
	Q6	2887	27.1	2819	23.0	2830	23.3	2803	22.3	2872	21.7	2917	21.3	3040	18.8	3125	18.1	3113	18.1	3119	17.1
Urbanization																					
	High	5463	19.8	5370	18.9	5345	18.8	5162	17.7	4992	17.0	5214	17.3	5734	15.5	5795	15.1	5796	14.8	6049	14.3
	Low	5792	33.8	5574	32.5	5570	33.1	5515	32.2	5572	30.7	5581	30.3	5658	26.7	5806	26.2	5779	25.5	5698	24.2

Q1, lowest ; Q6, highest. High urbanization area, administrative districts with a tertiary hospital or a stroke care unit; low urbanization area, the other area.

### 3.3 Prehospital Visit Time from the Final Admission for Treatment

**Fig. 3.**
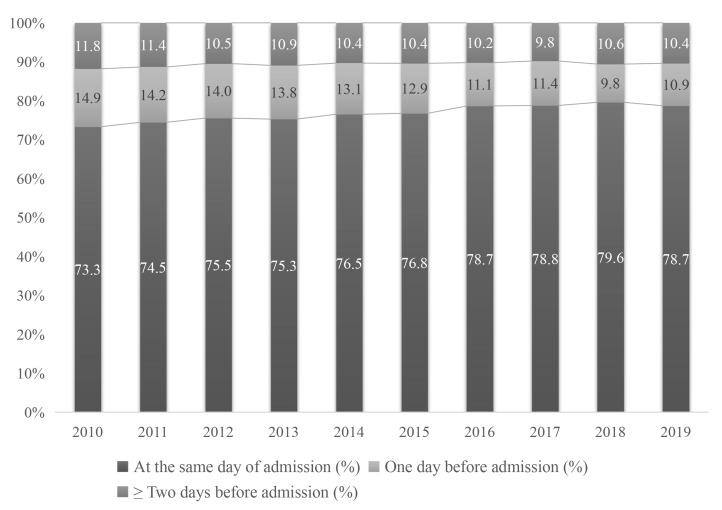
**Annual intervals between the prehospital visit and final admission for treatment from 2010 to 2019**.

Fig. 3 presents the time intervals from the prehospital visits to the final admission for treatment as three separate groups: (1) visiting on the same day of admission (zero days), (2) visiting at one day before admission (one day), and (3) visiting at least two days before admission (≥ two days). From 2010 to 2019, prehospital visits at zero days increased from 73.3% to 78.7%. Meanwhile, the proportion of patients with at least two days before admission improved slightly from 11.8% in 2010 to 10.4% in 2019.

## 4. Discussion

In this Korean study of claims data, 21.3% of acute stroke patients were found to have visited one or more hospitals before diagnosis. The annual rates of prehospital visits decreased over time. However, more than 10,000 patients yearly still visited a different medical institution to treat their acute symptoms, and the number of patients with prehospital visits did not reduce because of increased stroke incidence.

A systematic review of 123 papers published between 1981 and 2007 reported a wide range in prehospital delay times (mean hours, 1.2–98.8 hours; arrivals within three hours, 6%–92%) [2]. In the Report of Assessment for the Quality of Acute Stroke Care in Korea 2018, which analyzed data from stroke patients defined by ICD-10 codes, the median interval from onset to ED admission was 214 minutes (interquartile range [IQR], 65–844 minutes) [17]. According to EMS usage, the prehospital delay time showed a significant gap, i.e., 118.5 (IQR, 49–382) minutes for EMS users vs. 525 minutes (IQR, 163–1507.5) minutes for non–EMS users [17]. Although we could not measure the time from symptom onset to the ED admission in this study, a delay in arrivals could stem from not contacting the EMS, considering the rate of patients who visited more than one day before final admission for treatment (20.41%) in 2018 according to previous research [18].

Several studies of prehospital visits among stroke patients have focused on patient-related factors (e.g., recognized symptoms, awareness brought on by public education or campaigns, or prior experiences) [18, 19], system-related factors (i.e., triage by EMS or accessibility of EMS services, including due to geographical [e.g., distance from a stroke care units], temporal [e.g., traffic in rush hour], or socioeconomic factors [e.g., level of insurance coverage]) [8, 15, 20, 21]. Prehospital delays are multifactorial, triggered by both patient- and system-related factors [20]. This study added evidence of system-related factors, suggesting the cause of delay was due to visiting at least one hospital before ED admission.

Fortunately, the rate of prehospital visits has decreased over time. This gradual decline every year may result from public health education to increase stroke awareness by highlighting common symptoms and the time-critical nature of the disease [7]. The implementation of public education focused on teaching about stroke symptoms to facilitate the seeking of emergency care where appropriate has been recommended by the American Heart Association/American Stroke Association since 2013 [22]. About 69% of urban people aged at least 50 years old and 79% of people voluntarily participating in a nationwide stroke educational program performed by the Korean Stroke Society responded that they would call EMS to visit early as soon as possible [23, 24]. The Korean general population’s early stroke symptom recognition rate improved from 51.2% in 2017 to 61.7% in 2019. However, it decreased recently (54.2% in 2021) [25]. This study did not directly compare with the pre-and post-period of the COVID-19 outbreak. When the recent trends of prehospital visits were evaluated, we need to consider various factors related to the COVID-19 outbreak, such as changes in stroke incidence, awareness of disease, or EMS use [26]. During the COVID-19 outbreak, the proportion of direct visits to the ED of acute ischemic stroke patients through public EMS services increased. However, their prognosis was worse than that of pre-COVID-19 patients with delays at the prehospital delay [26]. Even with improvements in the public’s knowledge of the definition of stroke and its warning signs, there are several reasons that the rate of prehospital visits remains high. First, a relatively low barrier to medical use might have influenced the rate of prehospital visits to Korean medical services. Patients unable to identify their symptoms might contact their local or community doctor first rather than go directly to the ED [27]. Second, 59.2% of patients used EMS, according to the Report from the Epidemiology Research Council of the Korean Stroke Society, which was lower than the rates of other Organization for Economic Co-operation and Development countries (58%–79%) [28]. Third, the type of symptom(s) being experienced might influence the prehospital delay. For example, vague symptoms, such as sensory disturbances, vertigo, or dizziness, were associated with a prehospital delay, while the presence of more severe symptoms, such as confusion, decreased consciousness, or aphasia, was associated with early arrival [19, 29]. The American Heart Association “Face, Arm, Speech, Time to Call 911” campaign is based on recognizing weakness and aphasia or dysarthria [30]. The use of EMS may be affected by the nature of the symptoms recognized; in other words, there may be more significant EMS usage among patients with severe symptoms, such as weakness, aphasia, or an altered level of consciousness [31]. Although we did not survey the subjects’ initial symptoms, the high prehospital visit rate in young participants can be explained by an inability to predict stroke with no severe symptoms or to select an appropriate hospital.

It is interpreted that patients with higher income status showed a greater rate of prehospital visits in 2010, which can be explained that medical expenses incurred by prehospital visits are not a problem for high-income individuals [32]. On the other hand, low income was associated with a significant increase in prehospital delays in Nepal, which has not yet established a public health insurance system [33]. Previous research found no association between income status and prehospital delay in the middle- and high-income countries [6, 14]. Therefore, the unique association between income status and the rate of prehospital visits in Korea may have had an impact. A difference in the residential area may be associated with the rate of prehospital visits because patients with residences in low urbanization areas contacted the EMS less frequently (*p *= 0.009) and more frequently made prehospital visits (*p* = 0.020) without a different awareness of stroke or distance to the hospital [21]. We found a reversal in the trend of prehospital visits since 2016 according to sex. The reversed proportion of prehospital visits in female patients may have been due to the higher participation in public education or campaigns than male patients [23].

### 4.1 Limitations

First, we could not obtain the time data by prehospital visit, distance from a stroke unit, utilization of EMS, and stroke awareness because we obtained the visit information from the claims data based on the data available. Therefore, we did not compare the results with previous studies that reported delayed times [28]. Second, age, sex, and residence area were not adjusted, which may affect the trend. Due to claims data, family history of stroke or stroke-related laboratory data could not be surveyed. Third, the rate of prehospital visits may have been underestimated when a stroke was not suspected or another diagnosis, e.g., headache or dizziness, was considered. Lastly, our study results may not be generalizable to non-Korean stroke patients because we must consider the different medical service use factors, including EMS, health insurance status, and public education level.

### 4.2 Future Directions

Further research for the association between the number of prehospital visits and symptom severity regarding stroke will help to understand trends of prehospital visits. In addition, it will be necessary to identify the association between prehospital visits and clinical outcomes in acute stroke patients.

## 5. Conclusions

Prehospital visits in Korean stroke patients decreased from 25.1% in 2010 to 17.8% in 2019. However, more than 10,000 patients still visited other medical institutions before admission to treatment. Targeted policies to control prehospital delay by reducing the rate of prehospital visits among young patients and those who reside in low urban areas is needed.

## Data Availability

Raw data were generated at the National Health Information Database from the National Health Insurance Service in Korea. Derived data supporting the findings of this study are available from the corresponding author on request.

## Abbreviations

ED, the emergency department; EMS, emergency medical service; NHID, National Health Information Database; NHIS, National Health Insurance Service; ICD-10, International Classification of Diseases, 10th revision; CT, computer tomography; MRI, magnetic resonance image.
